# Cyberbully victimization and its association with residual depressive symptoms among clinically stable adolescents with psychiatric disorders during the COVID-19 pandemic: A perspective from network analysis

**DOI:** 10.3389/fpsyg.2022.1080192

**Published:** 2023-02-01

**Authors:** Xiao-Meng Xie, Hong Cai, Shu-Ying Li, Zong-Lei Li, Wu-Yang Zhang, Yan-Jie Zhao, Yao Zhang, Gabor S. Ungvari, Yi-Lang Tang, Fan He, Yu-Tao Xiang

**Affiliations:** ^1^The National Clinical Research Center for Mental Disorders and Beijing Key Laboratory of Mental Disorders, Beijing Anding Hospital and the Advanced Innovation Center for Human Brain Protection, School of Mental Health, Capital Medical University, Beijing, China; ^2^Unit of Psychiatry, Department of Public Health and Medicinal Administration, Institute of Translational Medicine, Faculty of Health Sciences, University of Macau, Taipa, Macao SAR, China; ^3^Centre for Cognitive and Brain Sciences, University of Macau, Taipa, Macao SAR, China; ^4^Department of Psychiatry, The First Affiliated Hospital of Zhengzhou University, Zhengzhou, China; ^5^Department of Psychiatry, Xiamen Xianyue Hospital, Xiamen, China; ^6^Department of Pediatric Development and Behavior, The Third Affiliated Hospital of Zhengzhou University, Zhengzhou, China; ^7^Section of Psychiatry, University of Notre Dame Australia, Fremantle, WA, Australia; ^8^Division of Psychiatry, School of Medicine, University of Western Australia / Graylands Hospital, Perth, WA, Australia; ^9^Department of Psychiatry and Behavioral Sciences, Emory University, Atlanta, GA, United States; ^10^Mental Health Service Line, Atlanta VA Medical Center, Decatur, GA, United States

**Keywords:** COVID-19, cyberbullying, victimization, psychiatric disorder, adolescent patients, network analysis

## Abstract

**Objective:**

This study examined the prevalence of cyberbullying and its relationship with residual depressive symptoms in this patient population during the COVID-19 outbreak using network analysis.

**Methods:**

This was a multicenter, cross-sectional study. Adolescent patients attending maintenance treatment at outpatient departments of three major psychiatric hospitals were included. Experience of cyberbullying was measured with a standard question, while the severity of Internet addiction and depressive symptoms were measured using the Internet Addiction Test and the Patient Health Questionnaire-9, respectively. The network structure of depression and cyberbully were characterized and indices of “Expected Influence” was used to identify symptoms central to the network. To identify particular symptoms that were directly associated with cyberbully, the flow function was used.

**Results:**

Altogether 1,265 patients completed the assessments. The overall prevalence of cyberbullying was 92.3% (95% confidence interval (CI): 90.8–93.7%). Multiple logistic regression analysis revealed that male gender (*p* = 0.04, OR = 1.72, 95%CI: 1.04–2.85) was significantly associated with higher risk of cyberbullying, while a relapse of illness during the COVID-19 pandemic was significantly associated with a lower risk of cyberbullying (*p* = 0.03, OR = 0.50, 95%CI: 0.27–0.93). In the network of depression and cyberbully, “Sad mood,” “Anhedonia” and “Energy” were the most central (influential) symptoms. Furthermore, “Suicidal ideation” had the strongest negative association with cyberbully followed by “Guilt”.

**Conclusion:**

During the COVID-19 pandemic, the experience of cyberbullying was highly prevalent among clinically stable adolescent psychiatric patients, particularly male patients. This finding should raise awareness of this issue emphasizing the need for regular screening and interventions for adolescent patients. Central symptoms (e.g., “Sad mood,” “Anhedonia” and “Energy”) identified in this study should be targeted in interventions and preventive measures.

## 1. Introduction

The Coronavirus Disease 2019 (COVID-19) pandemic has led to a huge loss of human life worldwide and has presented an unprecedented challenge to public health. It has dramatically altered the lifestyle and daily routine activities of people all over the world (Huang et al., 2020; World Health Organization, 2020). During the COVID-19 pandemic, psychiatric patients have been generally at higher risk of cross-infection due to their limited awareness of self-protection, poor self-restraint, and non-compliance with preventive public health measures (Gautam et al., 2020). Furthermore, due to widespread preventive measures including lockdowns, reduced face-to-face appointments and limited outpatient/inpatient services, psychiatric patients often had restricted access to mental health services (Xiang et al., 2020b; Kılınçel et al., 2021). Among different age groups affected by the COVID-19 pandemic, adolescent psychiatric patients are arguably one of the most vulnerable subpopulations that need special attention, because of the fear of infection, sudden school closures, and home quarantine in many areas during the COVID-19 pandemic (Constable et al., 2020), which may induce psychological distress and psychiatric disorders such as anxiety and depression, or worsen existing psychiatric symptoms.

During the COVID-19 pandemic, due to a range of public health measures, Internet use and online activities have increased dramatically (Dost et al., 2020) for both professional and personal purposes, particularly creating problems for inexperienced and unsophisticated adolescents (Hawdon et al., 2020). For instance, many adolescents could not concentrate on their study, and often moved their attentions from online class to internet games and/or online communications with others during the pandemic, which may be a contributing factor for increased risk of cyberbullying (Dong et al., 2020). In recent decades, incidents of cyberbullying of adolescents have been on the rise (Wolak et al., 2007; Chang et al., 2013). For instance, the Federal Bureau of Investigation (FBI) Internet Crime Complaint Centre (IC3) reported that cybercrimes in the US have quadrupled during the COVID-19 pandemic (England, 2020). Cyberbullying is defined as a variety of actions about intentional harassment and bullying, which harms the victim’s interest or reputation (Ivbijaro et al., 2021; Poyraz et al., 2021; Sartorius, 2021). It has three categories: first, written-verbal cyberbullying, such as sending nasty, threatening emails or messages, making fun of others in online chats, spreading gossip or insulting remarks about others online. Second, visual cyberbullying, such as taking embarrassing photos or videos and sharing them. Third, impersonation cyberbullying such as stealing someone else’s identity to reveal personal information or his/her financial situation (Tokunaga, 2010; Vivolo-Kantor et al., 2014; Jadambaa et al., 2019). The prevalence of cyberbully varies greatly across studies (4.8% − 72%) (Juvonen and Gross, 2008; Sourander et al., 2010; Kowalski et al., 2014; Hutson, 2016; Jadambaa et al., 2019) owing to the discrepancies in study settings, populations and assessment tools.

Cyberbullying is associated with a range of negative outcomes in victims, including physical comorbidities and psychological distress (Sampasa-Kanyinga et al., 2018; Al Qudah et al., 2020), impaired social functioning, poor quality of clinical care, and dissatisfaction with life (Diomidous et al., 2016; Hellfeldt et al., 2019). Therefore, to shed light on the impact of cyberbullying on adolescent psychiatric patients and to develop appropriate educational programs and preventive measures, it is important to examine the frequency and experience of cyberbullying in this vulnerable subpopulation during the COVID-19 pandemic. There have been no data on the pattern of cyberbullying in adolescent patients during the COVID-19 pandemic.

Network analysis is a novel statistical method in which mental health problems are viewed as systems of interacting symptoms that may give rise to each other as epiphenomena as a disorder progresses (Borsboom, 2017). Network analysis has the potential to map specific relationships between individual symptoms of a disorder and identify specific symptom targets for treatment (Fried et al., 2014). In the theory of network model, central nodes are the most influential symptoms of a disorder that could activate other symptoms and play a major role in causing the onset and/or maintenance of a syndrome.

This study examined the prevalence of cyberbully and explored its associated factors in clinically stable adolescent psychiatric patients in China during the COVID-19 pandemic. Previous studies using traditional statistical approaches based on total scores and/or cutoff values of standard scales found that cyberbully was significantly associated with the severity of depression (Landstedt and Persson, 2014; Zhang D. et al., 2020), but its associations with individual depressive symptoms are not clear. Therefore, we examined the inter-relationship between cyberbully and depressive symptoms using network analysis.

## 2. Materials and methods

### 2.1. Setting and sample

A multicenter, cross-sectional survey was carried out from April 29 to June 13, 2020 (i.e., in late stage of the COVID-19 outbreak) in outpatient departments at three major psychiatric hospitals in Beijing and Henan and Fujian provinces, which are located in the north, central and south of China and represent different geographical areas. Due to the risk of cross-infection, traditional face-to-face interview could not be conducted. Instead, following recent studies (Hao et al., 2020; Li et al., 2021), the WeChat-based Questionnaire Star program was used to collect data. The WeChat is a social communication application with more than 1 billion users. To be eligible, participants (1) were aged between 13 and 17 years that was used to define adolescents in participating hospitals; (2) had principal psychiatric diagnoses according to the International Statistical Classification of Diseases and Related Health Problems, 10th Revision (ICD-10) (World Health Organization, 1992); (3) were clinically stable as judged by their treating psychiatrists; the definition of clinical stability referred to patients as “clinically stable” whose psychotropic drug doses were changed by less than 50% in the past 3 months (Bowden et al., 2000; Bowden, 2005; Xiang et al., 2007). This has been the standard definition in use in clinical practice at the participating hospitals; (4) were able to understand the content of the assessment. All adolescents treated in outpatient departments of the participating hospitals during the study period were consecutively invited to participated in this study. All participating adolescents verbally agreed to participate in the survey and their guardians provided written informed consent. The study protocol was approved by the respective Institutional Review Boards at the participating hospitals.

### 2.2. Instruments

Data on demographic and clinical information were collected including gender, age, residence (urban or rural), number of siblings, severe physical diseases, principal psychiatric diagnosis, perceived study pressure, parent–child relationship, guardian’s income, use of mass media for COVID-19, daily physical exercises, concerns regarding COVID-19, difficulty seeing a psychiatrist, treatment adherence, and relapse of psychiatric illness during the COVID-19 pandemic.

There was no validated standardized scale on cyberbullying available in China during the study period. Following previous studies (González-Cabrera et al., 2018; Peng et al., 2019), experiences about cyberbully victimhood during the COVID-19 pandemic were asked with a single standard question with dichotomous responses (yes/no): “Have you ever received any offensive or abusive message/humiliating photos/being the victims of rumors/phishing through your mobile phone or the Internet during the COVID-19 pandemic?.” A response of “yes” was considered “experiencing cyberbullying.” The severity of Internet addiction was measured using the Chinese version of the Internet Addiction Test (IAT) (Young, 1998; Lai et al., 2013). The IAT consists of 20 items with each ranging from 0 (“rarely”) to 5 (“always”), with satisfactory psychometric properties (e.g., Cronbach’s α = 0.93) (Lai et al., 2013). The severity of depressive symptoms was evaluated with the nine items of the Patient Health Questionnaire-9 (PHQ-9) (Zhang et al., 2013). Each item was scored from 0 (not at all) to 3 (almost every day), with the total score of ≥5 indicating “having depression” (Wittkampf et al., 2007). The PHQ-9 has been validated in Chinese populations with a sensitivity of 0.89 and a specificity of 0.77 (Chen et al., 2015).

### 2.3. Data analysis

#### 2.3.1. Univariate and multivariate analyses

Data analyses were performed with SPSS, Version 23.0 (IBM Corporation, Armonk, NY, USA). The Kolmogorov–Smirnov test was used to examine the normal distribution of continuous variables. Comparisons between patients with cyberbully and those without in terms of socio-demographic and clinical characteristics were conducted with chi-square tests, two independent samples sample *t*-tests, or nonparametric Mann–Whitney U tests, as appropriate. To explore the independent associations between demographic and clinical variables and cyberbullying, all the variables with a *p*-value of <0.1 in univariate analyses were entered as independent variables in multiple logistic regression analysis, while victims of cyberbullying was the dependent variable. The significance level was set at 0.05 (2-tailed).

#### 2.3.2. Network structure

The network model was estimated using the R software (R Core Team, 2020). We computed polychoric correlations of all the PHQ-9 items and cyberbully item to investigate edges of the network model. We also estimated the Graphical Gaussian Model (GGM), a popular network model, with the graphic least absolute shrinkage and selection operator (LASSO) and Extended Bayesian Information Criterion (EBIC) model using the R package *‘qgraph’* (Epskamp et al., 2018). GGM is a pairwise Markov random field (PMRF) model used for interval or ordinal data, in which edges are interpreted as partial correlation coefficients. The network was visualized using the *‘qgraph’* package, where thicker edges represented a stronger relationship between nodes. We estimated the centrality index, Expected Influence (EI) of nodes, to determine the symptoms that were more central (influential) in the network model (Beard et al., 2016). To identify particular symptoms that were directly associated with cyberbully, the ‘flow’ function in R package ‘*qgraph’* was used (Epskamp et al., 2012).

#### 2.3.3. Network stability

Centrality stability was examined using the correlation stability coefficient (CS-coefficient). A CS-coefficient value above 0.25 indicates that observed network model results are stable, though traditionally, CS-coefficient values above 0.5 are preferable. A bootstrapped difference test was conducted to assess the robustness of node EIs and edges. Differences were significant between two nodes or two edges if zero was not included in the 1,000-bootstrap 95% confidence interval (CI). Edge accuracy was estimated with bootstrapped 95% CIs; a narrower CI suggests a more reliable network. These procedures were conducted using the package ‘bootnet’ v1.4.3 (Epskamp and Fried, 2020).

#### 2.3.4. Network comparison

Following previous studies (Lai et al., 2020; Zhang W. R. et al., 2020), the differences of network characteristics between male and female participants were compared, using the R ‘*NetworkComparisonTest’* package (Version 2.2.1) (van Borkulo et al., 2017) with 1,000 permutations. Differences in network structure (e.g., distributions of edge weights), global strength (e.g., total absolute connectivity among the symptoms), and each specific edge between subsamples (i.e., females vs. males) were also examined.

## 3. Results

A total of 1,570 adolescent patients were invited; 1,265 met study entry criteria and were included in the analyses, giving a participation rate of 80.6%; 1,168 (92.3%; 95%CI: 90.8–93.7%) experienced cyberbullying during the COVID-19 pandemic. The comparison of socio-demographic and clinical characteristics between cyberbully victim patients and those without it is presented in Table 1.

**Table 1:** Socio-demographic and clinical characteristics of clinically stable adolescent psychiatric patients.

Variable	Total		Non-Cyberbullied		Cyberbullied		Univariate analyses		
	(*N* = 1,265)		(*N* = 97)		(*N* = 1,168)				
	*N*	%	*N*	%	*N*	%	*χ* ^2^	df	*p*
Male gender	474	37.5	24	24.7	450	38.5	7.26	1	**0.007**
Rural residence	569	45.0	35	36.1	534	45.7	3.36	1	**0.07**
Only child at home	497	39.3	37	38.1	460	39.4	0.06	1	0.81
Major medical conditions	67	5.3	3	3.1	64	5.5	1.02	1	0.31
Principal psychiatric diagnosis							8.62	3	**0.04**
MDD	699	55.3	65	67.0	634	54.3			
BD	118	9.3	7	7.2	111	9.5			
ADHD	30	2.4	4	4.1	26	2.2			
Others	418	33.0	21	21.6	397	34.0			
Perceived study pressure							1.68	2	0.43
Low	187	14.8	14	14.4	173	14.8			
Fair	618	48.9	42	43.3	576	49.3			
High	460	36.4	41	42.3	419	35.9			
Relationship with parents							3.51	2	0.17
Good	465	36.8	29	29.9	436	37.3			
Fair	609	48.1	48	49.5	561	48.0			
Poor	191	15.1	20	20.6	171	14.6			
Concerns regarding COVID-19							1.78	2	0.41
Highly concerned	328	25.9	20	20.6	308	26.4			
Moderately concerned	697	55.1	59	60.8	638	54.6			
Not or minimal concerned	240	19.0	18	18.6	222	19.0			
Daily physical excise							0.32	2	0.85
Less than 30 min/day	932	73.7	72	74.2	860	73.6			
30–60 min/day	251	19.8	20	20.6	231	19.8			
More than 60 min/day	82	6.5	5	5.2	77	6.6			
Use of mass media for COVID-19							0.16	2	0.92
No or seldom	399	31.5	29	29.9	370	31.7			
Sometimes	578	45.7	46	47.4	532	45.5			
Often	288	22.8	22	22.7	266	22.8			
Difficulty seeing a psychiatrist during the COVID-19 pandemic							7.75	2	**0.02**
No or seldom	985	77.9	67	69.1	918	78.6			
Sometimes	240	19.0	23	23.7	217	18.6			
Often	40	3.2	7	7.2	33	2.8			
Treatment adherence during the COVID-19 pandemic							3.23	2	0.20
Poor	439	34.7	32	33.0	407	34.8			
Fair	172	13.6	19	19.6	153	13.1			
Good	654	51.7	46	47.4	608	52.1			
Relapse during the COVID-19 pandemic							12.32	2	**0.002**
No	550	43.5	27	27.8	523	44.8			
Symptomatic worsening, but no relapse	466	36.8	41	42.3	425	36.4			
Relapse	249	19.7	29	29.9	220	18.8			
Guardians’ income (RMB3000 and above/month)	814	64.3	68	70.1	746	63.9	1.52	1	0.22

	**Mean**	**SD**	**Mean**	**SD**	**Mean**	**SD**	***t/Z***	**df**	***p***
Age (years)	15.26	1.41	15.19	1.35	15.27	1.41	−5.65	1,263	0.57
PHQ-9 total	8.70	8.52	10.86	9.17	8.52	8.44	−2.30	–^a^	**0.02**
IAT total	41.43	19.13	47.57	21.79	40.92	18.82	3.30	1,263	**0.001**

a, Mann–Whitney U test; Bold values, <0.1; COVID-19, Coronavirus Disease 2019; MDD, Major depressive disorder; BD, Bipolar disorder; ADHD, Attention deficit hyperactivity disorder; PHQ-9, 9-item Patient Health Questionnaire; IAT, Internet addiction disorder; SD, standard deviation.

Univariate analyses revealed that cyberbully victims were more likely to be male (*p* = 0.007), rural residence (*p* = 0.07), had a principal psychiatric diagnosis other than major depressive disorder (*p* = 0.04), had difficulties seeing a psychiatrist (*p* = 0.02), relapsed during the COVID-19 pandemic (*p* < 0.01), had elevated PHQ-9 (*p* = 0.02), and higher IAT total scores (*p* < 0.01). Multiple logistic regression analysis found that male adolescent patients (*p* = 0.04, OR = 1.72, 95%CI: 1.04–2.85) had a higher risk of becoming cyberbully victims, while relapse of illness during the COVID-19 pandemic was significantly associated with a lower risk of being targeted by cyberbullying (*p* = 0.03, OR = 0.50, 95%CI: 0.27–0.93) (Table 2).

**Table 2:** Independent correlates of becoming a cyberbully victim by multiple logistic regression analysis.

Variables	Multiple logistic regression analysis			
	*p*	OR	95% CI	
			Lower	Upper
Male gender (ref. female)	**0.04**	1.72	1.04	2.85
Rural residence (ref, urban)	0.16	1.37	0.88	2.13
Principal psychiatric diagnosis				
MDD	–	1	–	–
BD	0.27	1.59	0.70	3.62
ADHD	0.11	0.39	0.12	1.22
Others	0.18	1.44	0.84	2.44
Difficulty seeing a psychiatrist during the COVID-19 pandemic				
No or seldom	–	1	–	–
Sometimes	0.26	0.74	0.45	1.24
Often	0.11	0.49	0.20	1.19
Relapse during the COVID-19 pandemic
No	--	1	--	--
Symptomatic worsening, but no relapse	0.11	0.65	0.39	1.10
Relapse	**0.03**	0.50	0.27	0.93
PHQ-9 total	0.73	1.01	0.97	1.04
IAT total	0.09	0.99	0.97	1.00

Bold values, *p* < 0.05; CI, confidential interval; OR, odds ratio; COVID-19, Coronavirus Disease 2019; MDD, Major depressive disorder; BD, Bipolar disorder; ADHD, Attention deficit hyperactivity disorder; PHQ-9, 9-item Patient Health Questionnaire; IAT, Internet addiction disorder. Study site has been controlled for as a covariate.

### 3.1. Network structure of depressive symptoms and cyberbully

**Figure 1:**
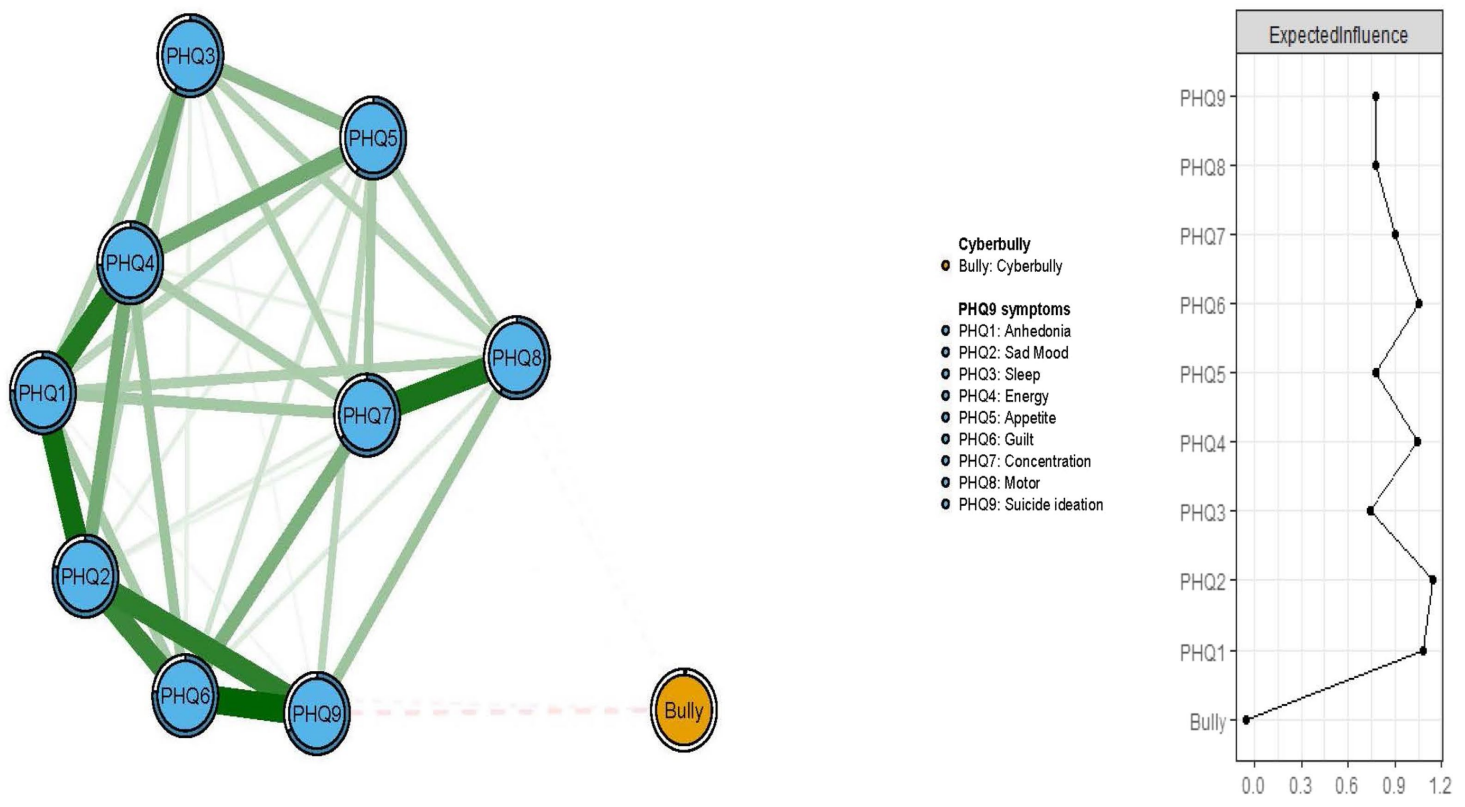
Network structure of cyberbully and residual depressive symptoms in clinically stable adolescent psychiatric patients.

Figure 1 presents the network structure of residual depressive symptoms and cyberbully. The model shows that the connection between nodes PHQ6 (“Guilt”) and PHQ9 (“Suicide ideation”) (average edge weight = 0.31) was the strongest positive edge, followed by the edges between nodes PHQ1 (“Anhedonia”) and PHQ2 (“Sad mood”) (average edge weight = 0.30), and between nodes PHQ1 (“Anhedonia”) and PHQ4 (“Energy”) (average edge weight = 0.27).

**Figure 2:**
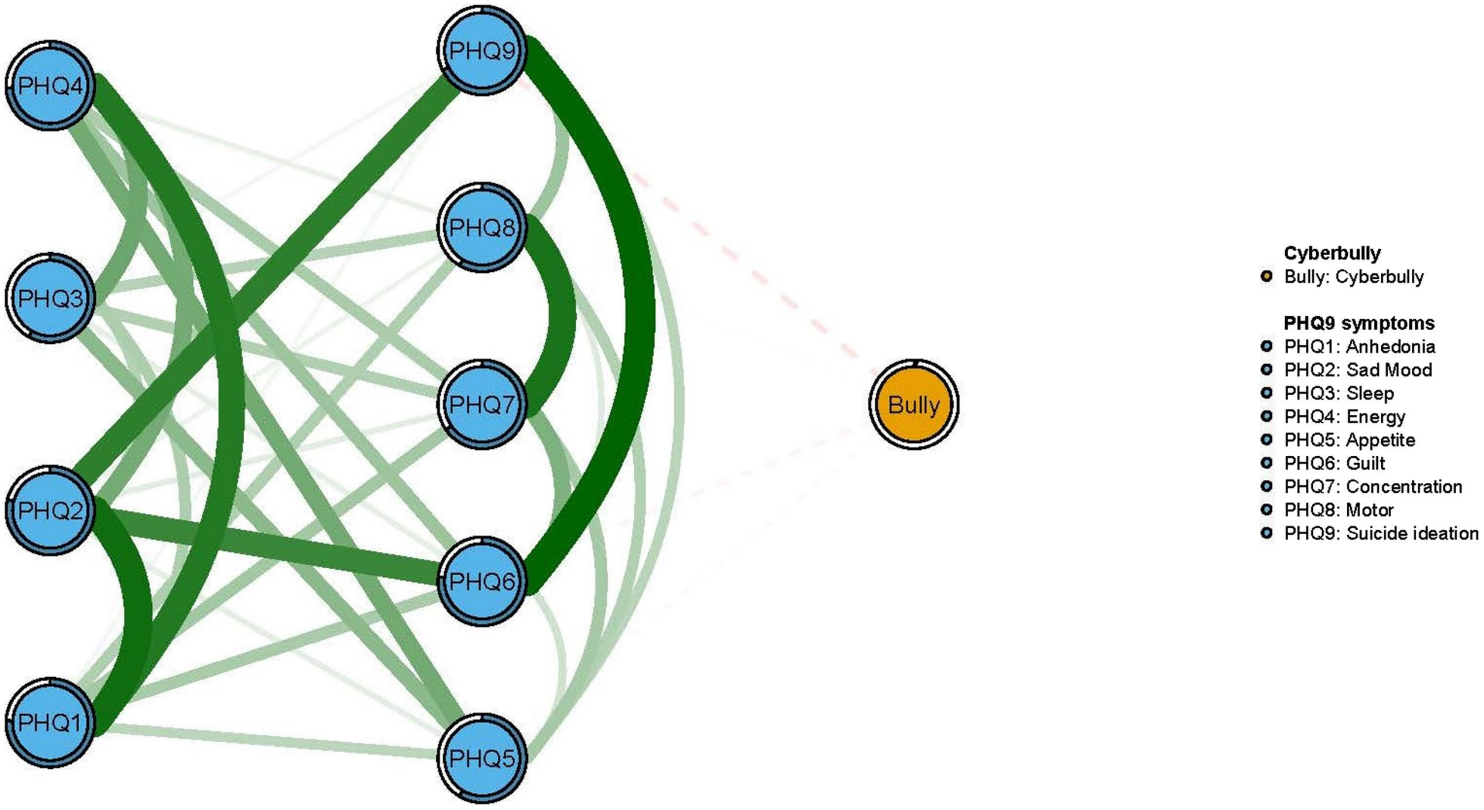
Flow network of cyberbully and residual depressive symptoms.

In terms of EI in the network model, the node PHQ2 (“Sad mood”) had the highest EI centrality, followed by nodes PHQ1 (“Anhedonia”) and PHQ4 (“Energy”) (Figure 1), indicating that these three symptoms were the most influential ones for understanding the network model of depression and cyberbully in clinically stable adolescents with psychiatric disorders. In addition, we found that PHQ9 (“Suicide ideation”) had the strongest negative association with cyberbully in the flow network model, followed by the PHQ6 (“Guilt”) (Figure 2).

**Figure 3:**
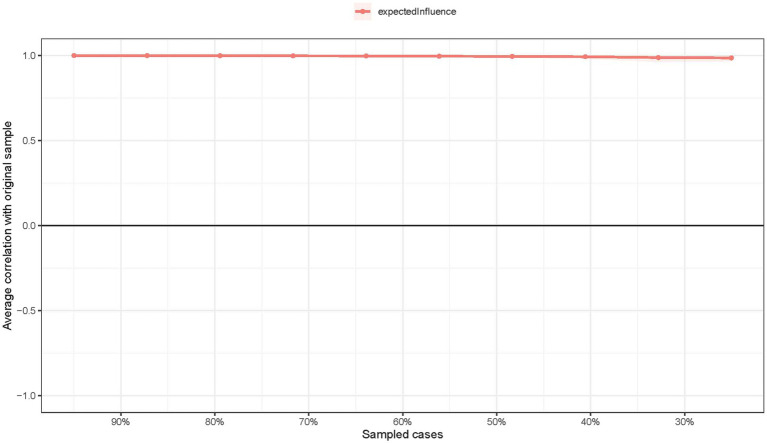
The stability of centrality indice using case-dropping bootstrap.

The EI centrality had an excellent level of stability (i.e., CS-coefficient = 0.75; 95%CI: 0.672–1), and results of bootstrapped differences tests for edge weights showed that most comparisons between edge weights were statistically significant, indicating that the network model were reliable and stable (Figure 3).

### 3.2. Network comparison tests for gender

The comparison of network models between female and male adolescents did not show significant differences in network global strengths (4.22 in female and 4.22 male participants, respectively; *S* = 0.14, *p* = 0.84), and edge weights (*M* = 0.13, *p* = 0.74; Supplementary Figures S1, S2).

## 4. Discussion

To the best of our knowledge, this was the first study that estimated the prevalence of cyberbully suffered by adolescent psychiatric patients during the COVID-19 pandemic. Based on a large sample of adolescent psychiatric outpatients, a strikingly high rate (92.3%, 95% CI: 90.8–93.7%) of cyberbullying was found in this vulnerable population during the COVID-19 pandemic.

While there are no similar data available to compare this finding in this patient population, the figure of 92.3% is much higher than the rates of cyberbullying in the general population of adolescents. For example, in an online survey conducted in adolescents aged between 12 and 17 years in the US, 72% of the participants experienced at least once cyberbullying (Juvonen and Gross, 2008). A meta-analysis revealed that among Australian children and adolescents, the lifetime prevalence of experiencing cyberbullying was 7.02% (95% CI: 2.41–13.54%) (Jadambaa et al., 2019). A survey of secondary school students in Guangzhou, China, found that 44.5% of participants experienced cyberbullying in the previous 6 months (Rao et al., 2019). In another survey of college students in Hong Kong 68% of participants were subjected to cyberbullying (Leung et al., 2018).

Cyberbullying in adolescents has shown an increasing trend particularly in psychiatric patients. A recent study in the US found that 20% of adolescent psychiatric inpatients experienced cyberbullying (Saltz et al., 2020). Similarly, 14–27% of Australian children with psychiatric disorders were victims of cyberbullying (Islam et al., 2020). The rate of cyberbullying was 14.6% in adolescents with high-functioning Autistic Spectrum Disorder (Hu et al., 2019), and 19.1% in Attention-Deficit Hyperactivity Disorder (Yen et al., 2014). The discrepancy between studies in terms of the prevalence of cyberbullying could be partly due to different sampling, study setting, assessment methods and cultural factors (Guangming Online, 2019).

Patient- and COVID-19-related factors contributed to the high prevalence of cyberbullying in clinically stable adolescent psychiatric patients in this study. Many clinically stable adolescents still exhibit residual symptoms (Duffy et al., 2009; Chan, 2012), and have poor cognitive and social functioning (Bora et al., 2008) and psychotropic drug-induced side effects (Leucht et al., 2012). All these factors could impair online communication with others and invite cyberbullying. In addition, the lockdown, mandatory quarantine and related restrictions during the COVID-19 pandemic greatly changed people’s life and created psychological problems such as anxiety and irritability (Xiang et al., 2020a), which could have also increased the risk of the addiction to Internet and cyberbullying. Furthermore, due to the limited knowledge about SARS-CoV-2, particularly during the early stage of the pandemic, misinformation and rumors about COVID-19 and quarantine measures were widespread in social media, such as Tik Tok, WeChat, Facebook, Twitter, and Instagram, which also led to communication conflicts and cyberbullying.

The findings on gender differences in cyberbullying have been inconsistent with studies reporting male (Yang et al., 2006; Calvete et al., 2010; Chang et al., 2013; Leung et al., 2018), or female (Smith et al., 2006; Sourander et al., 2010) predominance, or no gender difference (Li, 2006; Smith et al., 2008; Livingstone et al., 2011). In this study, male adolescents experienced more frequent cyberbullying, probably because they are more likely to engage in “risky” online activities, including online video games, surfing the “dark web,” or inviting strangers as friends to their social networks, all of which increase the likelihood of becoming a victim of cyberbullying (Patchin and Hinduja, 2010; Fanti et al., 2012; Costello et al., 2016; Lapierre and Dane, 2020). It was expected that the risk of cyberbullying would be higher in adolescent patients who experienced symptom worsening or relapse during the COVID-19 pandemic, as most psychiatric symptoms interfere with communication with others. Surprisingly, relapse during the pandemic was significantly associated with a lower risk of cyberbullying, probably because relapsed patients received more parental supervision or had to restrict their online activities for other reasons. Interestingly, according to concept of bullying circle, if a target has other characteristics such as low empathy or a high level of reactive aggression, as frequently happens when adolescent psychiatric patients relapse, adolescents may turn their anger or fear onto others and thus become a perpetrator (Moreno and Radovic, 2018). The symptoms of adolescent psychiatric patients also appear as atypical;“irritability” was a common symptom in this population. When adolescent patients relapse, abnormalities in both reward and threat processing underlie the clinical presentation of irritability, which includes a greater propensity toward affective (e.g., frustration and anger) and behavioral (e.g., motor activity and aggression) responses (Brotman et al., 2017). Therefore, when relapsed, adolescent could transfer themselves from a bullying target to perpetrator thereby preventing cyberbullying. Personality traits of bullying perpetrators and targets have also been well characterized. The main personality trait of impulsivity has been found to be associated with conduct problems including bullying in adolescents (Frick and Dickens, 2006).

“Sad mood” was one of the most central symptoms identified in the depression-cyberbully network. Similar findings were reported in previous network analysis studies on depressive and anxiety symptoms in adult psychiatric patients (Beard et al., 2016; Park and Kim, 2020), adults with depression (Fried et al., 2016; Kaiser et al., 2021), and children and adolescents with depression and anxiety (McElroy et al., 2018). Mentally ill adolescents were often unable to attend classes, found it difficult to see psychiatrists or take their medicine, and engage in outdoor activities due to strict public health measures during the COVID-19 pandemic, which could increase the likelihood of emerging sad mood (Loades et al., 2020; Liu et al., 2021).

Anhedonia was another central symptom in the depression-cyberbully network. As a depressive symptom and also a trait vulnerability to depression (Di Nicola et al., 2013), anhedonia is associated with low sensitivity to reward (Huys et al., 2013; Liu et al., 2014) and reduced activation in the ventral striatum in response to pleasant or rewarding stimuli (Der-Avakian and Markou, 2012; Keller et al., 2013). Lack of energy was also a central symptom in the depression-cyberbully network in this study. Lockdown and other restriction measures led to sudden school closures, a switch to online teaching, cancelation of examinations, and outdoor sports and group physical activities stopped in many areas during the COVID-19 pandemic, all of which could indirectly contribute to the development of lack of energy and fatigue (Chen et al., 2020; Zhou et al., 2020; Wright et al., 2021).

Experiencing bullying is a risk factor of suicidal ideation among adolescents (Hinduja and Patchin, 2010a; Iranzo et al., 2019; Kim et al., 2019). Unexpectedly, the flow network model revealed a negative association between cyberbully and suicide ideation in this study. In China, cyberbully among mentally ill adolescents has been an ongoing concern and can often easily be identified by parents and teachers (Huang and Chou, 2010; Hinduja and Patchin, 2010b). Therefore, timely protective measures and support could be provided to adolescents suffering from cyberbully offsetting the risk of negative feelings and related problems such as suicidal ideation. This could also explain the negative association between cyberbully and guilty feeling identified in flow network analysis in this study.

The merits of this study include the multicenter study-design, the large sample and the use of network analysis. However, several limitations of the study should be considered. First, because of the cross-sectional study design, the causality between cyberbullying and other variables could not be established. Moreover, the data were collected from April 29 to June 13, 2020; therefore, the findings could not be generalized to other stages of the pandemic. Second, data on cyberbully prior to the COVID-19 outbreak were not collected, thus the findings could not be compared to the frequency of cyberbully experienced by the same patient population in the pre-COVID-19 era. Third, similar to most previous studies (Ang and Goh, 2010; Sourander et al., 2010; Duarte et al., 2018; González-Cabrera et al., 2018), data on cyberbullying were collected *via* self-report, therefore the possibility of recall bias could not be excluded. In addition, information obtained by self-report on cyberbullying may be unreliable when it comes to sensitive topics, thus collateral information from others (e.g., teachers, guardians, and peers) will be needed in future studies. Further, psychiatrically ill, emotionally unstable adolescents may have exaggerated or underplayed their experiences with cyberbullying. Fourth, cyberbullying was recorded as a binary variable (presence/absence), therefore, the patterns and associated factors of different subtypes / severity of cyberbullying could not be examined. Fifth, potentially important factors related to cyberbullying, such as family and social support, guardianship of cyberspace, level of computer skills, and health-related quality of life, were not recorded. Sixth, the data collected by consecutive sampling method were used to construct network model, which could limit the representativeness of the sample.

Due to the devastating consequences of cyberbullying, effective measures targeting the central symptoms (e.g., Sad mood, Anhedonia and Energy) should be taken to prevent and reduce its risks. Parents could protect their children from cyberbully by teaching them to use safe Internet tools, showing them how to protect their personal data, limiting their time on social media, and asking children to report cyberbullies to parents and/or teachers (Gordon, 2021). Adolescents should be able to protect themselves by ignoring cyberbullying messages, to resist the urge to retaliate or respond, to take a screenshot of cyberbullying messages and save it as an evidence, to report and block cyberbullying, and to talk to parents when experiencing cyberbullying and feeling uncomfortable or unsafe about some information from the internet (TeensHealth, 2018; Ditch the Label, 2019).

In conclusion, cyberbullying was common in clinically stable adolescent psychiatric patients in in three main regions of China during the COVID-19 outbreak. The findings highlight the need to improve the awareness of cyberbullying in this vulnerable population. Central symptoms (e.g., “Sad mood,” “Anhedonia” and “Energy”) identified in this study should be vigorously treated while preventive measures for cyberbully in this population such as social support and active interventions targeting cyberbullying including the use of strong security measures (Hawdon et al., 2020) (e.g., antivirus software and firewalls), and regular screening are urgently needed.

## Data availability statement

There are stringent restrictions in making the research dataset of the clinical studies publicly available. Readers and all interested researchers may contact Y-TX (xyutly@gmail.com) to apply for exemptions from the participating institutions if appropriate.

## Ethics statement

The studies involving human participants were reviewed and approved by Beijing Anding Hospital. Written informed consent to participate in this study was provided by the participants’ legal guardian/next of kin.

## Author contributions

FH and Y-TX: study design. Z-LL, HC, FH, S-YL, Y-JZ, W-YZ, and YZ: data collection, analysis and interpretation. X-MX, Y-LT, and Y-TX: drafting of the manuscript. GU: critical revision of the manuscript. All co-authors approved the final version for publication.

## Funding

The study was supported by the University of Macau (MYRG2019-00066-FHS; MYRG2022-00187-FHS), Sci-Tech Innovation 2030 - Major Project of Brain science and brain-inspired intelligence technology (2021ZD0200600), Beijing Scholar 2021 (No.: 063), and Beijing Hospitals Authority Clinical Medicine Development of special funding support (XMLX202128).

## Conflict of interest

The authors declare that the research was conducted in the absence of any commercial or financial relationships that could be construed as a potential conflict of interest.

## Publisher’s note

All claims expressed in this article are solely those of the authors and do not necessarily represent those of their affiliated organizations, or those of the publisher, the editors and the reviewers. Any product that may be evaluated in this article, or claim that may be made by its manufacturer, is not guaranteed or endorsed by the publisher.
