# Supplementary material for: Cyberbully victimization and its association with residual depressive symptoms among clinically stable adolescents with psychiatric disorders during the COVID-19 pandemic: A perspective from network analysis
Source: Front Psychol. 2023 Feb 1;13:1080192. doi: 10.3389/fpsyg.2022.1080192 (PMC9929464; doi:10.3389/fpsyg.2022.1080192)

**Supplementary materials**

Figure S1. Estimated network model for the association between cyberbully and residual depressive symptoms in females and males.


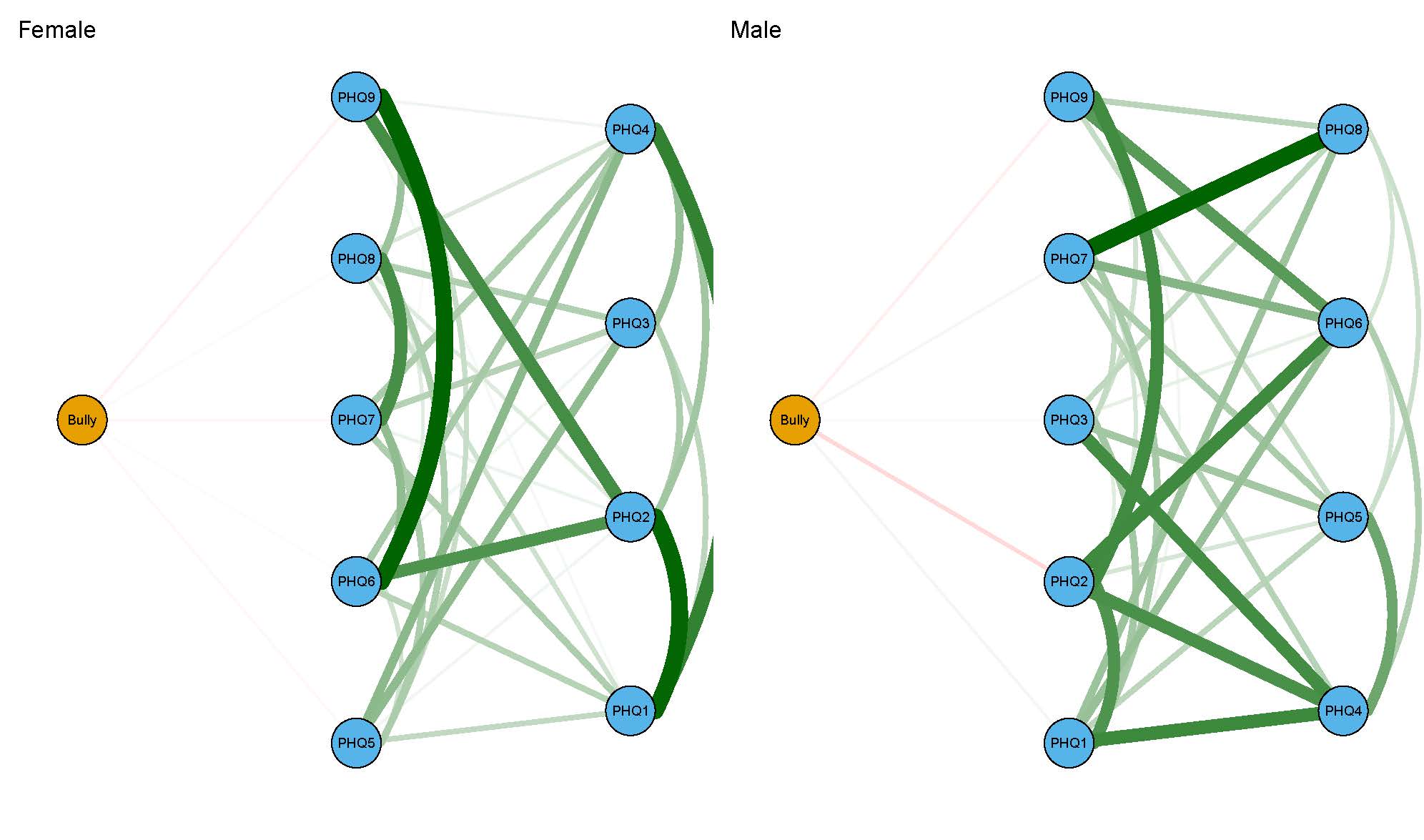


Figure S2. Comparison of network properties between females and males.


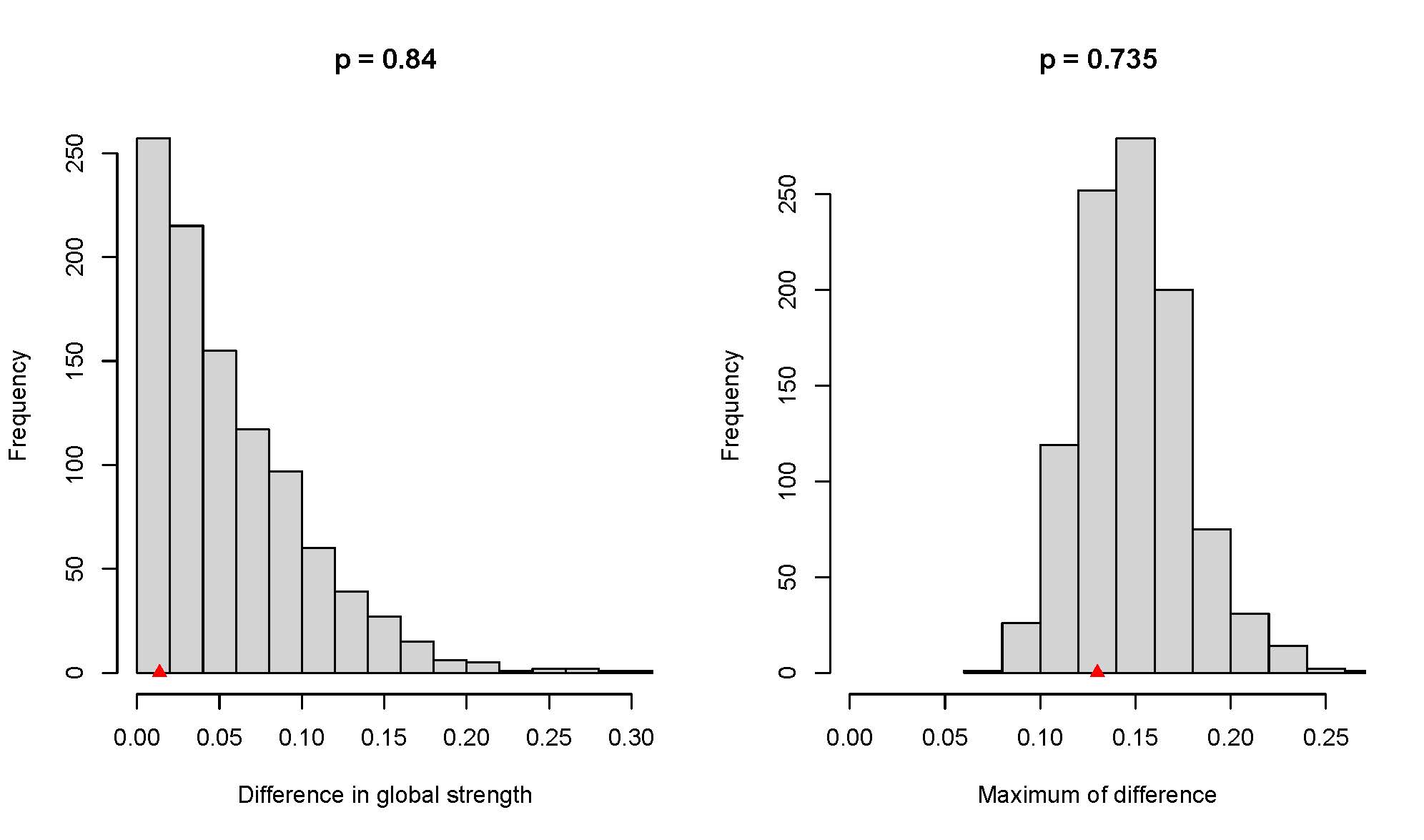

Supplement: Supplementary file 1 [file Data_Sheet_1.docx]
